# The use of a metaphyseal sleeve total knee replacement as primary treatment for Schaztker VI tibial plateau fracture

**DOI:** 10.1093/jscr/rjac561

**Published:** 2022-12-20

**Authors:** Matthew McSorley, Monu Jabbal, Phil Walmsley

**Affiliations:** Department of Orthopaedics, Royal Infirmary of Edinburgh, Edinburgh EH16 4SA, UK; Department of Orthopaedics, Royal Infirmary of Edinburgh, Edinburgh EH16 4SA, UK; Department of Orthopaedics, Royal Infirmary of Edinburgh, Edinburgh EH16 4SA, UK

**Keywords:** Tibial-plateau fracture, Schatzker VI, revision knee arthroplasty, metaphyseal sleeve, trauma arthroplasty

## Abstract

Traditional treatment of tibial plateau fractures is with open reduction and internal fixation, or external fixation in severely displaced and comminuted fractures. Total joint arthroplasty for unreconstructable hip fractures is a successful and widely accepted treatment; however, such surgery for tibial plateau fractures is not a common practice. We present two cases of highly comminuted schaztker VI tibial plateau fractures in patients over the age of 65. Both patients had a metaphyseal sleeve revision knee arthroplasty as delayed primary treatment. Both patients have had excellent clinical and radiographical results at 6 months and 5 years follow-up, respectively. We present the first description in the literature of this implants use for bone loss as a result of trauma. There is growing evidence that total joint arthroplasty is an effective treatment in tibial plateau fractures, in particular for elderly patients who may be at high risk of failure from internal fixation.

## INTRODUCTION

We present two cases (both male patients over 65 years) who sustained a Schaztker VI tibial plateau fracture and underwent definitive treatment with a porous coated metaphyseal sleeve total knee replacement (TKR) as the primary procedure. We believe this is the first report of the use of this technique in the literature. There is a growing body of evidence to support TKR as the primary treatment for some elderly patients with highly comminuted injuries that would be at high risk of fixation failure. We discuss the cases, rationale for the use of TKR in this context, evidence for TKR and proposed advantages of using a porous-coated metaphyseal sleeve system.

## CASE 1

**Figure 1:**
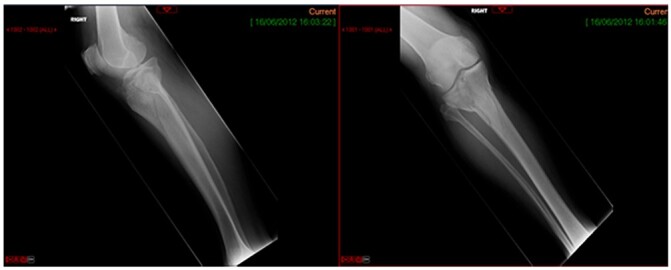
Plain radiograph AP and lateral.

**Figure 2:**
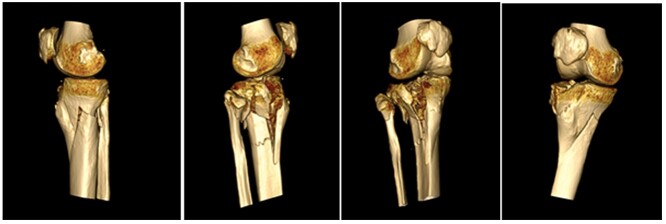
CT reconstruction.

**Figure 3:**
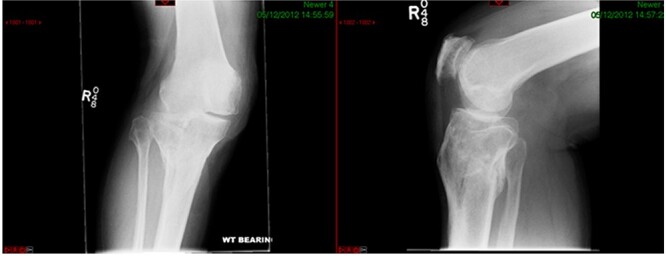
Healed fracture position.

**Figure 4:**
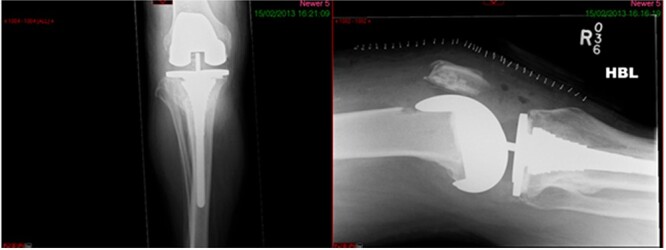
Immediate post-op radiograph.

**Figure 5:**
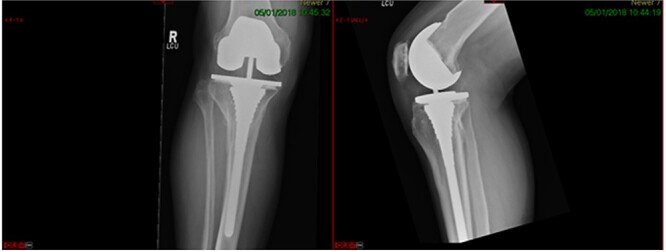
7-year post-op radiograph.

 A 68-year-old gentleman who was diagnosed with a Schatzker type VI fracture having fallen 3 m from a ladder (Figs 1 and 2). The injury was closed and neurovascularly intact. The patient was monitored for signs of compartment syndrome. He rapidly developed significant fracture blisters that persisted for over 2 weeks despite regular dressing care. He was deemed too high risk for acute operative intervention because of a high risk of wound complications. Following a multidisciplinary team discussion, he was managed in a cast then a knee brace, mobilising non weight bearing for 12 weeks, at which point the fracture was seen to be healing in a valgus malalignment. The patient was allowed to partially weight bear and received physiotherapy. He was reviewed regularly until fracture had united (Fig. 3). At 8 months post-injury, the patient underwent total knee arthroplasty utilizing a Depuy Synthes (Warsaw, In) PFC Sigma TC3 tibial metaphyseal sleeve and stem, and femoral component without sleeve or stem (Fig. 4). The patient was reviewed post-operatively in clinic at 6 months, the wound had fully healed and the range of motion was 0°–120°. The patient’s pain was significantly reduced, and they were independently mobile. They were kept under annual review and seen at 5 years post-operatively with an Oxford Knee score of 35; the patient used no walking aids and was pain free at rest or during normal walking, with the radiograph demonstrating good osseointegration. The latest follow-up at 8 years revealed no radiographical change in implant (Fig. 5).

## CASE 2

**Figure 6:**
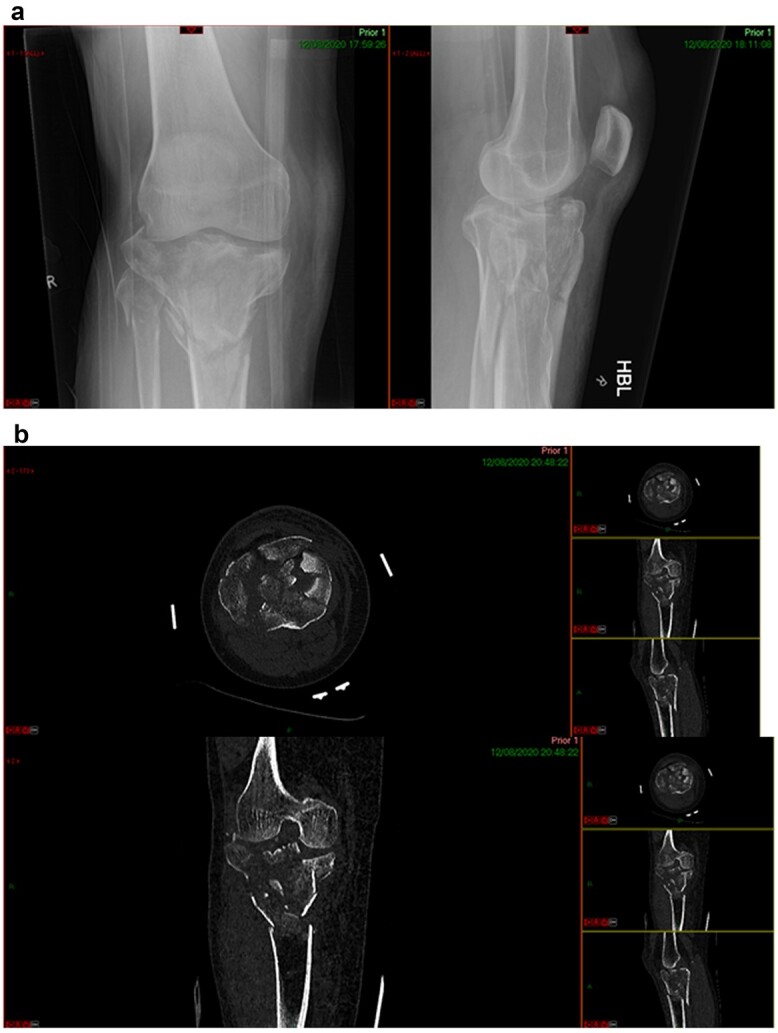
(**a**) Post-injury radiograph, (**b**) post-injury CT scan.

**Figure 7:**
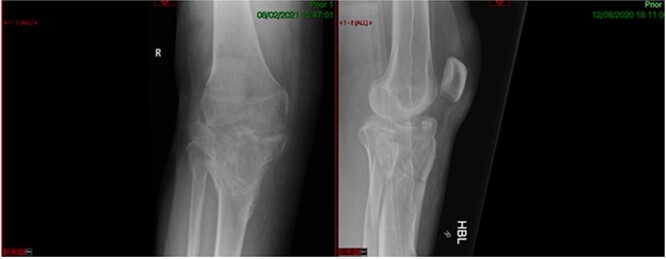
Pre-operative healed fracture.

**Figure 8:**
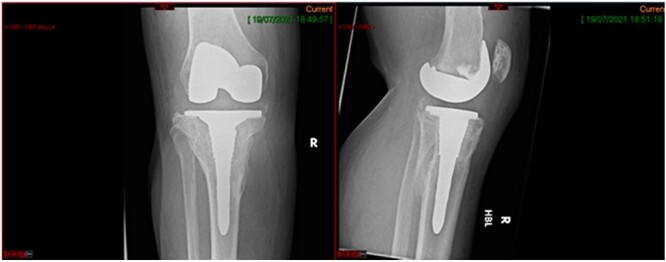
6-month post-operative knee replacement.

This case report follows a 67-year-old gentleman who sustained a Schatzker VI fracture of his right tibia after falling 2 m from a ladder (Fig. 6). The injury was closed and neurovascularly intact; he was managed initially in a neighbouring health board with a bridging external fixator because of the high degree of comminution. He was referred to the current institution; and at a routine review 3 weeks post-injury, it was noted that a pin had fractured. The external fixator was removed, and he was converted into an above knee cast. He progressed to a Sarmiento cast and then a range of motion brace at 12 weeks. Partial weight bearing was permitted, and he received physiotherapy. He was kept under regular review and the fracture had united (Fig. 7); he was noted to have a range of movement of 10°–60° with healed pin site wounds. At 8 months post-injury, he underwent total knee arthroplasty utilizing a posterior stabilised Depuy Synthes (Warsaw, In) Attune revision tibial metaphyseal sleeve and stem, and femoral component without sleeve or stem. The patient had an uncomplicated recovery and kept under routine review in the outpatient clinic. At 6 months’ review, the patient had a range of motion of 0°–105°, fully healed wound and reported a significant improvement in pain. The patient used no walking aids, and the Oxford Knee score was 40 (Fig. 8).

## DISCUSSION

Stevenson *et al*. describe the demographic of those who suffer tibial plateau fractures. For younger patients, the mechanism of injury tends to be high-energy trauma. Low-energy falls are a more common mechanism of injury in older patients. In all, 55–70% of tibial plateau fractures involved the lateral plateau with approximately 10–23% involving the medial plateau and 10–30% involving both tibial condyles [1].

Schatzker VI (bi-condylar) tibial plateau fractures present a significant challenge to surgeons, and they are often highly comminuted with associated ligamentous injury. Management options include open reduction and internal fixation (ORIF) and circular frames, with many patients suffering residual pain and dysfunction after the treatment has been completed. Infection risk is relatively high because of swollen and friable soft tissues compromised by pin sites or plates. The Canadian Orthopaedic Trauma Society reported only 12–30% of patients in their prospective randomized control trial returned to all previous activities, with significant residual limb specific and general health deficits at 2 years post-injury [2].

Traditional practice in treating those with severe tibial plateau fractures is the management of ORIF or external fixation [3], with TKR performed as a secondary procedure after failure or complication [4]. Thought is now being given to the use of employing TKR as the initial treatment for these patients, as described in the cases above.

### Challenges of ORIF in elderly versus proposed advantages of TKR

 Complications post ORIF result from a combination of poor bone quality, metaphyseal bone comminution and friable soft tissue envelope. All of these predispose patients to increased risk of fixation failure and wound complications [5]. There is also often a requirement to protect weight bearing for weeks after the surgery, have regular wound review because of healing issues and a need for long-term monitoring because of the risk of post-traumatic osteoarthritis [6]. Ali *et al*. performed a retrospective review of 42 consecutive patients with surgically treated tibial plateau fractures, and reported a 79% fixation failure rate in patients with marked osteoporosis. They also showed that complex fragmentation and nonadherence to weight-bearing instructions were significantly correlated with a loss of reduction [7].

Malunion and post-traumatic osteoarthritis can necessitate conversion into TKA. Wasserstein *et al*. report that 7.3% of patients underwent conversion to TKR from ORIF at 10 years, with age, female sex, co-morbidity and increased comminution of fracture attributing to this [8]. Secondary TKR can be technically challenging surgery and are at a risk of higher complication rates and poorer functional outcomes [9]. The reasons for this are multifactorial: previous scar, joint stiffness, hardware, ligament insufficiency and marked malalignment. Furthermore, elderly patients may be too comorbid to receive a second demanding surgery and rehabilitation [1].

Like a hip replacement being used in the context of a proximal femur fracture, there is growing interest in TKR as primary treatment of a proximal tibia fracture. This should only be considered when the risk of failure of fixation is high, because of highly comminuted injuries, bone loss or patient comorbidity. The main proposed advantage is a lower failure rate with fewer (or no) subsequent surgeries. When performed acutely, there is immediate stability and weight bearing, leading to less complications of reduced mobility such as thrombosis, chest infection and deconditioning [10]. Not all cases are suitable for acute surgery and some may be delayed primary procedure. TKR, in this scenario, is not without its own problems and particular mention is made to the need for revision-type implants and increased levels of constraint for possible ligamentous injury [6]. Revision of such a TKR would be a challenging operation; however, because of the patient demographic chosen, the implant should survive the entirety of the patient’s life.

### Evidence of TKR

A systematic review by Wong *et al*. examined a patient group of 105 who underwent primary TKR for tibial plateau fracture [10]. The mean age was 72.7 years, M:F 43:62 with a 39 months’ follow-up. The impressive overall Knee Society Knee score was 86 ± 6 and the Function score 65 ± 14. The mean knee flexion was 108°±10°. The vomplication rate was 15%. This comprised of eight patients who needed revision (three wound issues, two periprosthetic fractures, one infection, one aseptic loosening, one residual cement). Other complications were superficial wound infection (three), one deep vein thrombosis and one patient with mild genu valgum.

Tapper *et al*. performed a systematic review of 197 patients who underwent primary TKA for tibial plateau fracture. The mean patient age range was 68–86, and the mean follow- up was 28 months. In sum, 108 patients were classified as per AO: 7 A-type (1%), 70 B type (65%) and 31 C type (39%); 71 patients were classified as per Schatzker: 28 II-type (39%), 15 III-type (21%), 14 IV-type (20%), 11 V-type (15%) and 2 III-type (4%). Two studies reported mean Oxford Knee scores of 29.5 and 35.7. The remaining studies had a mean global Knee Society score of 153 (SD 15). The mean flexion was 107°. Out of 163 patients, 12 reported 10 complications (6.1%), resulting in six revisions (3.6%). Complications were infection (four), periprosthetic fracture (three), DVT (one), loosening (one) and stiffness (one).

Only two studies used in both these reviews have a small control group of ORIFs; however, both reports improved flexion and functional outcome scores in the TKR group. Both also report lower complication and re-operation rates in the TKR group. The overall quality of current literature is low; however, based on the limited evidence, TKR appears to be a useful treatment in elderly patients. The complication and revision rates after primary TKR are lower than secondary TKR, however still higher than elective TKR.

### Use of metaphyseal sleeve for bone loss

Metaphyseal sleeves can be an effective option for the management of bone loss in TKR. They are geometrically stepped in design and coated with titanium beads allowing for bone in-growth. The sleeve can then be combined with a Morse taper junction to either the femoral or tibial component. Surgical technique involves reaming the host bone to accept compaction broaches which are sequentially increased in size until rotational and axial stability is achieved during on-table testing [11]. The sleeves can be used with or without an intramedullary stem, which, in itself, can be cemented or press-fit [12]. If there is significant deformity in the bone, the sleeve can be used with no stem to gain the optimal alignment. Advantages of this technique are rotational stability because of the stepped design, highly osseointegrative coating and immediate loading of metaphyseal bone to prevent stress shielding. Bloch *et al*. reported favourable results using this implant for revision TKR for any cause, with 97.8% survival at 10 years of 73 implants. They report five failures (defined as re-revision for any reason), four for infection and one for instability [13].

Martín-Hernández *et al*. reviewed the use of metaphyseal sleeves as the primary implant for TKA after post-traumatic knee arthritis in 25 patients with a mean age of 64 years. Their focus on the metaphyseal zone and its fixation is because of it being less osteoporotic in post-traumatic arthritis. It is also more vascularized than the epiphyseal zone and not compromised by defects resulting from injury or its complications. The mean time between osteosynthesis of the fractures reviewed and TKA was 32.2 months, and in the follow-up, simple AP, lateral and full weight-bearing X-rays were used to assess possible subsidence of the components and signs of loosening. The mean follow-up was 79 months. A large improvement in all clinical scales (KSS, KSFS, WOMAC pain index, WOMAC stiffness and WOMAC function score) was seen. The mean physical and mental SF12 scores also improved. All patients were allowed immediate loading. The radiological follow-up showed all alignment in the interval to be optimum and all components were well fixed.

## CONCLUSION

We present two cases of male patients over 65 years of age who fell from ladders sustaining Schaztker VI tibial plateau fractures. Both patients were deemed at a high risk of failure of fracture fixation; thus, they underwent delayed primary TKR after the fracture had time to heal. After an uneventful period of rehabilitation, both patients returned to walking unaided and with minimal to no pain. At 7 years’ follow-up, the first patient had no radiographical signs of failure and was still pain-free. Schaztker VI tibial plateau fractures can be a very challenging injury for surgeons to manage, with many patients experiencing residual pain and functional deficit. The body of literature for primary TKR (acute or delayed) is growing to suggest it is an effective treatment in some patients, and, at the same time, the porous coated metaphyseal sleeve is achieving excellent outcomes as an implant for managing bone loss in revision TKR. The authors suggest the implant has been an effective choice for this challenging injury, which is the first description of this technique in the literature.

## CONFLICT OF INTEREST STATEMENT

None declared.

## FUNDING

None.
